# nail: software for high-speed, high-sensitivity protein sequence annotation

**DOI:** 10.1101/2024.01.27.577580

**Published:** 2024-01-30

**Authors:** Jack W. Roddy, David H. Rich, Travis J. Wheeler

**Affiliations:** 1 R. Ken Coit College of Pharmacy, University of Arizona, Tucson, Arizona, USA;; 2 Department of Computer Science, University of Montana, Missoula, Montana, USA

## Abstract

“ Fast is fine, but accuracy is final. ”

-- Wyatt Earp

**Background:**

The extreme diversity of newly sequenced organisms and considerable scale of modern sequence databases lead to a tension between competing needs for sensitivity and speed in sequence annotation, with multiple tools displacing the venerable BLAST software suite on one axis or another. Alignment based on profile hidden Markov models (pHMMs) has demonstrated state of art sensitivity, while recent algorithmic advances have resulted in hyper-fast annotation tools with sensitivity close to that of BLAST.

**Results:**

Here, we introduce a new tool that bridges the gap between advances in these two directions, reaching speeds comparable to fast annotation methods such as MMseqs2 while retaining most of the sensitivity offered by pHMMs. The tool, called nail, implements a heuristic approximation of the pHMM Forward/Backward (FB) algorithm by identifying a sparse subset of the cells in the FB dynamic programming matrix that contains most of the probability mass. The method produces an accurate approximation of pHMM scores and E-values with high speed and small memory requirements. On a protein benchmark, nail recovers the majority of recall difference between MMseqs2 and HMMER, with run time ~26x faster than HMMER3 (only ~2.4x slower than MMseqs2’s sensitive variant). nail is released under the open BSD-3-clause license and is available for download at https://github.com/TravisWheelerLab/nail.

## Introduction

### Profile hidden Markov models for high sensitivity

The dominant method for accurate annotation of biological sequences is sequence database search, in which an unknown sequence is classified by aligning it to sequences in an established database. This alignment-based approach of annotating sequences has historically been associated with the Smith-Waterman algorithm (Smith et al., 1981) and fast heuristics such as BLAST (Altschul et al., 1990). In the years since the introduction of BLAST, profile hidden Markov models (pHMMs: Krogh et al. (1994); Durbin et al. (1998); Eddy (1998)) have been shown to produce superior sequence search sensitivity (Karplus et al., 1998; Krause et al., 2024).

Much of the sensitivity of pHMMs is due to their natural representation of profiles – when a collection of sequence family members is used to train the model, a pHMM captures the position-specific letter and gap frequencies inherent to the family. Profile representation of a family of sequences allows for improved search sensitivity relative to search using a collection of individual sequences (Gribskov et al., 1987; Eddy, 2011; Krause et al., 2024), and these families also enable faster annotation time when sequences can be compared to a single family profile rather than the family’s constituent members. This pair of benefits has driven the development and use of databases of sequence families and accompanying pHMMs all across bioinformatics, e.g. (Mistry et al., 2021; Mi et al., 2019; Gibson et al., 2015; Grazziotin et al., 2016; Storer et al., 2021; Huerta-Cepas et al., 2019).

Perhaps less appreciated is the fact that pHMM-based software is typically more sensitive than BLAST even when aligning to a database of individual sequences rather than profiles (Wheeler and Eddy, 2013; Steinegger and Söding, 2017; Frith, 2023; Krause et al., 2024). Unlike other alignment methods that compute just a single highest-scoring alignment (akin to a maximum probability Viterbi alignment (Viterbi, 1967) in pHMM terminology Durbin et al. (1998)), pHMMs enable computation of support for homology based on the sum of the probabilities of *all* alignments via the Forward/Backward (FB) algorithm (Rabiner, 1989; Krogh et al., 1994). Posterior probabilities resulting from FB also enable greater alignment accuracy (Holmes and Durbin, 1998; Do et al., 2005; Frith, 2023) as well as improved mechanisms for addressing composition bias and determining alignment boundaries (Eddy, 2008).

Computing FB is computationally expensive – to align a pair of sequences, FB requires completion of a dynamic programming matrix with size determined by the product of the sequence lengths, with each matrix cell requiring additional calculations to capture the sum of alignment probabilities (see Eddy (2011) for discussion). HMMER3 introduced a pipeline in which most candidates are never subjected to expensive FB analysis, thanks to a series of earlier filter stages. In common use cases, the first filter of HMMER3 (called MSV) consumes ~70% of HMMER’s run time, while FB consumes ~20% of time and is primarily responsible for large memory usage due to the quadratic-sized dynamic programming matrix required for recovering the alignment. FB dominates run time in cases of queries with high length or large numbers of true matches, and becomes the primary run time bottleneck in the event of improved speed for the earlier filter phases (Anderson and Wheeler, 2023).

### Algorithms for high speed

Recent years have seen remarkable speed gains for sequence alignment methods, including those targeting alignment of highly-similar sequences (Langmead and Salzberg, 2012; Li, 2013; Kim et al., 2019; Edgar, 2020; Li, 2021; Sahlin, 2022; Li, 2023) and those reporting BLAST-like sensitivity in the context of high sequence divergence (Steinegger and Söding, 2017; Buchfink et al., 2021). We focus here on MMseqs2 (Steinegger and Söding, 2017), a profile alignment tool that achieves exceptional speed gains relative to BLAST. The speed of MMseqs2 is primarily due to two innovations in its analysis pipeline. First, an optimized lookup table is used to restrict alignment computation to only involve matches with two very short high scoring length-k matches; these are extended to compute an ungapped alignment filter like that used in HMMER3. Next, MMseqs2 avoids the FB alignment step entirely, simply computing a highest-scoring alignment and using that as the basis of reported results. This approach produces impressive speed gains, and benefits from the advantages of position-specific scores, but misses out on the benefits of the more robust FB algorithm (Frith, 2023), resulting in modest loss in sensitivity relative to pHMM search (Krause et al., 2024). Another search tool, DIAMOND (Buchfink et al., 2021), has also demonstrated excellent speed, but its sensitivity does not appear to rival that of MMseqs2 (Krause et al., 2024).

### A hybrid pipeline for high-speed and sensitive alignment

**Figure 1.**
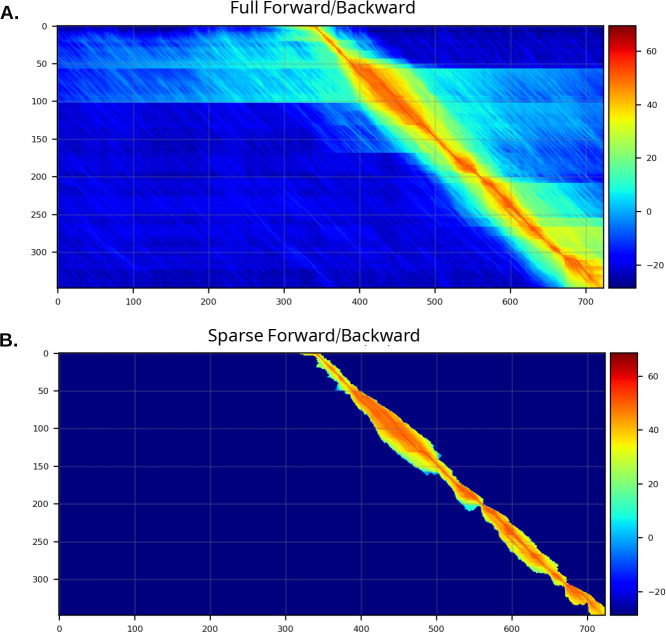
Sparsely filled Forward/Backward matrix capturing most of the probability mass. Top panel (A) shows heatmap of scores per cell in the Match State matrix for the sequence Q01LW8_ORYSA aligned to (the model for its matching family, DAO (FAD dependent oxidoreductase); bottom panel (B) shows the sparse set of (non-blue) cells that make up the cloud used for computing sparse Forward/Backward. The model positions are aligned along the y-axis and the sequence positions are aligned along the x-axis.

Here, we describe a sequence search pipeline that utilizes the MMseqs2 software suite to rapidly identify candidate sequence matches, then employs a fast FB heuristic to improve alignment sensitivity. The fast heuristic limits search space in the FB dynamic programming (DP) matrix to a high-probability cloud, as demonstrated in Figure 1, and results in calculations that closely approximate the results of the full FB algorithm, while providing a substantial reduction in space requirements and run time. The sparse FB implementation, along with downstream analyses making use of the resulting sparse posterior probability matrix, are based on methods in HMMER3, but are implemented from scratch in the Rust programming language, with the aim of creating a modern and stable codebase for reduced runtime and memory requirements of highly-sensitive sequence annotation. The software, called nail (for nail is an alignment inference tool), is released under an open (BSD 3-clause) licence; source code is available at https://github.com/TravisWheelerLab/nail and is hosted on the official Rust package registry at https://crates.io/crates/nail.

In the following sections, we demonstrate the efficacy of nail’s sparse FB implementation, demonstrate the impact of the overall pipeline on speed and sensitivity of sequence search, and provide a thorough description of its implementation.

## Results

The primary innovation of nail is the development of an approximate method that reduces the time and memory required for computation of the Forward and Forward/Backward (FB) algorithms for pHMMs, along with downstream analyses that are based on posterior probabilities resulting from FB (including creation of an alignment). The approach is a close cousin to the X-drop heuristic used in BLAST: start with a seed that establishes a region of interest within the DP matrix, and expand DP calculations out in both directions until pruning conditions are met – details are provided in the Methods section. Figure 1 presents a single example of the reduced computation required by nail’s sparse Forward/Backward for a relatively short alignment of one Pfam-based pHMM against a sequence belonging to the family. Seeds for nail’s FB heuristic are acquired by running MMseqs2 as a subroutine for candidate identification.

We begin by describing the data used for evaluation, then demonstrate the space-pruning efficacy of nail’s Cloud Search approach. We then show that annotation with nail significantly improves accuracy over maximum probability alignment, while adding only a small amount of processing time. Scripts and notes to reproduce benchmarking results can be found at https://github.com/TravisWheelerLab/nail-benchmarks.

### Benchmarks

#### Pfam domain benchmark

Assessment was performed primarily using a benchmark created with software (*create-profmark*) available in the HMMER3 release (Eddy, 2011). The benchmark consists of 1,339 families from Pfam-A v35.0 (Mistry et al., 2021) that could be split into a training and test set such that the test set contained at least 10 sequences and no training-test pair of sequences shares greater than 25% identity. The training set defines a multiple sequence alignment for each family, and we refer to the collection of training families as the query. For each family, sequences from the group not included in the training set were down-sampled such that at most 30 sequences were used for the family and no two sequences were *>*50% identical; this left 25,688 total sequences, which serve as the test set. Each true test sequence was embedded in a larger unrelated sequence, to simulate the sub-sequence nature of the protein domains in Pfam; specifically, unrelated sequences were produced by sampling from uniprot_sprot (2023_05), shuffling, then splicing the true test sequence into the middle of the shuffled sequence. This set of sequences containing true positives was supplemented with 2 million additional sequences sampled and shuffled as above, but with no embedded matches. By construction, this benchmark contains cases that are highly difficult for sequence alignment tools to recognize (train and test sequences are less than 25% identical), in order to emphasize differences in sensitivity. Note that the benchmark does not include reversed sequences, as these are prone to producing an excess of unexpected positives due to the surprising distribution high scores when aligning sequences to their reversals or even reversals of their homologs (Glidden-HandgisandWheeler,2023). For more details on benchmark construction method and philosophy, see (Eddy, 2011).

#### Long protein data set

Alignment with Pfam models represents a common use case for sequence alignment, but one that involves relative short sequences – the median Pfam domain length is just over 300. The purpose of nail’s sparse Forward/Backward implementation is to avoid calculation over a full quadratic-sized dynamic programming matrix, and longer sequences are the ones that suffer most from this quadratic scaling; we therefore performed some tests using sequences on the longer end of the protein sequence length distribution. Specifically, we captured 6 pairs of long sequences from Uniprot (Table 1), and performed experiments to assess time and space efficiency along with approximation accuracy. For each pair, one sequence was designated the *query* and the other the *target*.

#### Analysis pipeline – a sketch

As a first step, the nail pipeline runs MMseqs2 search, which rapidly produces a set of candidate query/target pairs by performing k-mer-based seed selection followed by fast local alignment. nail runs the standard MMseqs2 search pipeline (with a few parameters adjusted as in Table 2): (i) a k-mer match stage identifies candidate matches based on the presence of two co-diagonal length-k matches with score above a threshold score; (ii) a parameterized number of above-threshold paired k-mer matches are extended to capture only those candidates with good-scoring ungapped alignments, then (iii) surviving candidates are aligned to produce the single highest-scoring gapped alignment for each candidate query/target pair. After running MMseqs2 search, nail retains all results with P-value less than 0.01. The first and last positions of each surviving MMseqs2 alignment are mapped to corresponding cells (i.e. target and query positions) in a hypothetical FB alignment matrix. Using the mapped cells as a starting point, a heuristic search algorithm (Cloud Search) identifies a contiguous subset of FB matrix cells with non-negligible probability. Within this reduced set of matrix cells, nail then completes a sparse variant of Forward/Backward, producing an overall alignment score along with position-specific posterior probabilities that positions are aligned; these posterior probabilities are used to compute a composition bias score adjustment along with the final sequence alignment. See Methods for more details.

### Sparse Forward/Backward reduces computation, is a good approximation

To evaluate nail’s sparse Forward/Backward method, we tested the extent to which it reduces the number of computed cells, as this directly impacts time and space utilization. We also measured how well the sparse analysis approximates alignment scores computed using full Forward/Backward.

**Figure 2.**
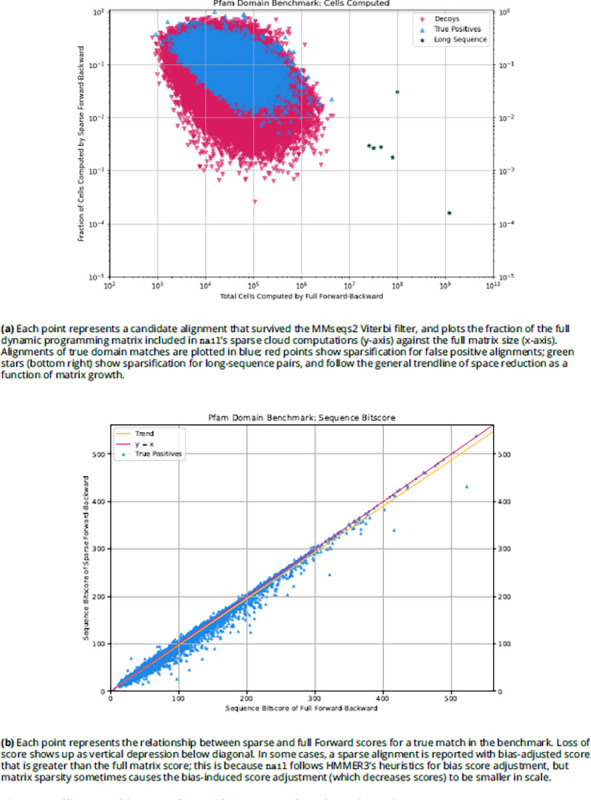
Efficacy and impact of sparsifying Forward/Backward matrix.

To analyze search space reduction, we computed the percentage of the full quadratic search space that is explored by the sparse approach. In Figure 2A, matrix sparseness (y-axis) is plotted against matrix size (x-axis – the product of the lengths of the query pHMM and target sequence). Reduction in search space is modest for alignments of shorter sequences; this is not surprising, as the total size of the matrix is not particularly large, so that even a modestly-wide band around the maximum-scoring alignment will consume much of the full analysis space. For longer sequences (for example with a length-400 model aligned to a length-2500 protein, creating a matrix of size 10^6^), nail’s sparse method often restricts the total number of computed cells to 1% or less of the full size of the matrix. Note that the sparsification is slightly greater on average for alignments involving false positive matches. Though nail’s implementation is not SIMD vectorized as in HMMER3 (Farrar, 2007; Eddy, 2011), the dramatic reduction in computed cells results in notable speed gains (see below). Figure 2B shows, for true positives from the domain benchmark, that the Forward score computed on the sparse matrix typically closely matches the score computed by Forward on the full matrix.

### Recall as a function of false annotation

We used the Pfam-based benchmark described above to assess the accuracy gains achieved with the Forward/Backward algorithm relative to MMseqs2 alignment, and to measure the efficacy of nail’s sparse implementation in retaining these gains. Each of the 1,339 query alignments was used to search for matching family members in the test database containing 25,688 true-embedded sequences mixed with 2 million simulated sequences. An alignment was considered to be ‘true positive’ if at least 50% of the length of an embedded target sequence was covered by an alignment with the query from the same family. A hit that entirely matched shuffled sequence was defined as a ‘false positive’. Alignments between a query and target of differing families were treated as neutral (ignored) rather than being penalized, since it is not possible to ensure lack of homology across assigned family labels.

**Figure 3.**
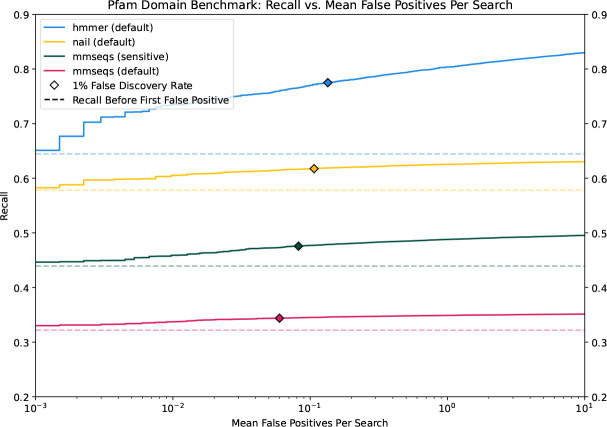
Recall as a function of false annotation rate. The protein domain benchmark consists of 25,688 true target sequences from 1,339 Pfam families, mixed with 2 million shuffled sequences from Uniprot. nail and HMMER were each tested with default parameters; MMseqs2 was tested with both default and sensitive (-s 7.5 --max-seqs 1000) settings.

Figure 3 presents recall (fraction of all true positives that are recovered at a specific E-value cutoff) as a function of false annotation rate (number of false positive matches per query with that E-value or better). For each tested method, all resulting alignments were gathered together and sorted by increasing E-value, so that a recall curve can be plotted. HMMER3’s hmmsearch tool was run with default settings (‘-E 10’), and establishes a sensitivity target; since hmmsearch can produce multiple ‘domain’ alignments for a matched query-target pair, the domain with the best score (lowest E-value) was retained. Curves are plotted for MMseqs2 with default and sensitive (‘-s 7.5 --maxseqs 1000’) settings, and indicate a large sensitivity loss relative to pHMM annotation with HMMER. nail closes this gap, particularly at low false positive levels, producing near-HMMER sensitivity with MMseqs2-like speed (see below).

The horizontal dashed lines in Figure 3 represent the recall before the first false positive for each tool, which we refer to as *recall-0* and is equivalent to the measure primarily reported in (Steinegger and Söding, 2017) and (Buchfink et al., 2021). All tools show at least several percent gain in recall beyond that first false positive, with HMMER showing the steepest recall gains. As implemented, nail essentially re-scores candidate matches produced by MMseqs2. To establish an upper bound on the recall gains possible with sparse Forward/Backward, nail includes an option to compute the *entire* Forward/Backward matrix for all candidates reported by MMseqs2 stage (‘--full-dp’). The corresponding curve is not shown here because it is essentially identical to that of the sparse implementation in nail. This demonstrates that loss of recall in nail relative to HMMER is due to limitations in candidates passing the initial filter, not failure of the sparse method, and highlights the value of future developments in fast candidate identification. We note that another high-speed annotation tool, DIAMOND (Buchfink et al., 2021), was omitted from analysis due to much lower benchmark sensitivity, in agreement with Krause et al. (2024).

Note: this analysis accentuates the difference in real world performance of the tools because the benchmark is constructed to consist exclusively of hard-to-find matches. Furthermore, the performance gap may also be overstated due to the fact that the benchmark is built from Pfam sequences, which themselves were partly gathered using HMMER. Even so, the analyses agree with other observations of superior pHMM sensitivity (Steinegger and Söding, 2017; Krause et al., 2024).

### Exploring the tension between speed and accuracy

**Figure 4.**
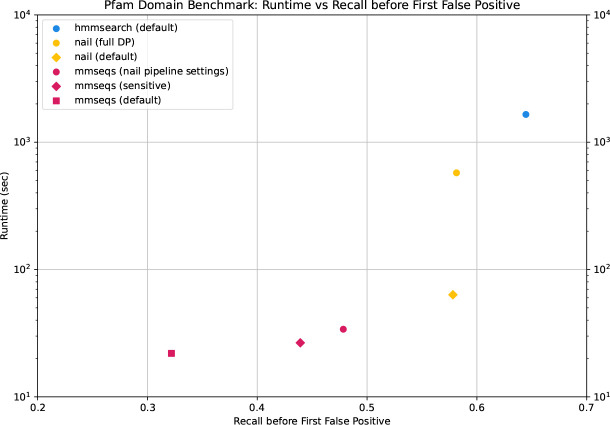
Run time vs. recall. Pfam-based benchmark was searched with tool variants to demonstrate performance-runtime tradeoffs. These include MMseqs2 variants (default; sensitive: -s 7.5 --max-seqs 1000; nail pipeline settings: --k-score 80 --min-ungapped-score 15 --max-seqs 1000), HMMER3’s hmmsearch (default), and nail variants (default; --full-dp). All tools were run with 8 threads.

Assessment of sequence annotation methods must consider the tradeoff between speed and sensitivity. In doing so, it is helpful to summarize the full recall curves from Figure 3 with a simple statistic. Here, we use the value *recall-0*, which is computed as the fraction of planted positives assigned an E-value better than the best-scoring false positive. This summary statistic is easy to interpret and generally agrees with relative ordering of methods in analyses such as Figure 3. Figure 4, plots run time (y-axis) and *recall-0* (x-axis) for annotation of the Pfam-based benchmark described above – an idea tool will produce a point that is low (fast) and to the right (sensitive). We view these results as a conservative estimate of the speed benefits of the sparse Forward/Backward approach, because the Pfam-based domain sequences are often quite short – the relative speed/recall tradeoff is expected to be increasingly in favor of sparse Forward/Backward for longer sequence elements (see Figure 2).

Figure 4 includes results of searching with HMMER3, which produces the highest *recall-0* values at the cost of ~62-fold increase in run time relative to sensitive MMseqs2. Recall and times for MMseqs2 default and sensitive are shown, along with values for MMseqs2 as parameterized when used within the nail pipeline (see Table 2). Meanwhile, nail recovers more than half of MMseqs2’s lost sensitivity, while increasing run time only ~2.4-fold. The full matrix variant of nail is also plotted, to demonstrate the speed boost achieved with sparse alignment, with essentially no loss in recall. A large majority of the sensitivity difference between nail and HMMER3 is the result of aggressive candidates filtering by the k-mer match stage in MMseqs2, suggesting that an alternate ultra-fast alignment seed detection method is warranted.

## Methods

### MMseqs2 as a prefilter for nail

The first step in the nail pipeline is to identify a collection of promising query-target candidates, along with alignment matrix positions that will serve as seeds for sparse matrix calculations. nail identifies candidates by running the MMseqs2 ‘search’ command with two non-default parameters (see Table 2). This produces a maximal-scoring alignment and E-value for each reported query-target pair. The E-value is a measure of significance of an alignment computed by internally adjusting the alignment’s P-value by the size of the search space (the P-value indicates, for an alignment with score *s*, the probability of a non-homologous pair of sequences producing score ≥ *s*). Echoing the filtering strategy used in HMMER3, the nail pipeline converts MMseqs2 E-values into P-values (by inverting the database size adjustment), then filters out candidates with P-Value *>* 0*.*01 (i.e. 1% of non-homologous query-target pairs are expected to pass the filter).

#### Mapping the MMseqs2 profile to a pHMM

Ideally, the previous step would provide landmarks (begin/end cells) in the pHMM alignment matrix for each identified candidate query-target pair. Because the alignment results correspond to an MMseqs2-style profile, and those profile positions do not necessarily map to the HMMER3-style pHMM positions used in nail’s Forward/Backward alignment, nail must map MMseqs2 profile position to the corresponding HMMER3 pHMM position. This is accomplished by performing an alignment of each MMseqs profile against the consensus sequence generated from the corresponding HMMER3 pHMM, using the MMseqs ‘search’ tool. The resulting alignment is used to map between the two profile representations through a linear scan.

### Default implementation of the Forward/Backward algorithm

To prepare for discussion of a sparse alignment implementation, we first describe the standard implementation of the Forward/Backward algorithm for aligning a query profile HMM (or sequence) to a target sequence. Input to the algorithm consists of:

- An alphabet Σ of size k(k=20 for the amino acid alphabet).
- A length-*n* target sequence $T=t_{1},t_{2},\ldots ,t_{n}$, with all $t_{j}\in \Sigma$.
- A query pHMM Q defined by a collection of values organized around three core states for each of m positions:
  - Match states (M) emit residues (letters) from Σ with a position-specific distribution, and during alignment are used to associate (match) a residue $t_{j}$ from T to a position $q_{i}$ in Q;
  - Insert states (I) emit residues in between match-state residues, and during alignment allow some residues in T to not correspond to positions in Q (to lie between matched residues). In principle, position-specific insertion emission probabilities are legal, but nail follows the common convention of employing a single emission distribution for all insert states (which matches the background distribution);
  - Delete states (D) are silent states (no emission) that, in alignment, allow some positions in Q to be deleted (not represented) in T.
  - Note: though this description introduces the query as a pHMM, nail is capable of searching with a single sequence. A single sequence will correspond to a pHMM in which emission probabilities are not position-specific, but instead depend simply on the observed residue at each position. Transition probabilities are uniform.

In support of these states, Q is described by two matrices (see Durbin et al., 1998 for more detail):

1. For each position i, emissions of match state $M_{i}$ are defined by a vector $q_{i1},q_{i2},\ldots ,q_{ik}$, where a value $q_{ic}$ corresponds to the model’s probability of observing residue c at position i.
2. A transition matrix captures the probability of transitioning from one state to another in sequential positions (transitions between D and I states are not included):
$$t\left(M_{i},M_{i+1}\right),t\left(M_{i},D_{i+1}\right),t\left(M_{i},I_{i}\right),t\left(I_{i},I_{i}\right),t\left(I_{i},M_{i+1}\right),t\left(D_{i},D_{i+1}\right),t\left(D_{i},M_{i+1}\right)$$

With this input, the Forward algorithm fills in three (m+1)(n+1) matrices, $F^{M},F^{I}$, and $F^{D}$, one for each state. The value stored at a cell (i,j) in a state’s matrix corresponds to all ways of aligning the first j letters of T with the first i model positions, ending in that state. After initializing $F_{0,0}^{M}=F_{0,0}^{D}=$ $F_{0,0}^{I}=0$, the remaining matrix cells are computed via the recurrence equations:

$$c=t_{j}$$

$$F_{i,j}^{M}=q_{ic}\cdot sum\left\{\begin{array}{c} F_{i-1,j-1}^{M}\cdot t\left(M_{i-1},M_{i}\right),\\ F_{i-1,j-1}^{I}\cdot t\left(I_{i-1},M_{i}\right),\\ F_{i-1,j-1}^{D}\cdot t\left(D_{i-1},M_{i}\right) \end{array}\right.$$

$$F_{i,j}^{I}=sum\left\{\begin{array}{c} F_{i,j-1}^{M}\cdot t\left(M_{i},I_{i}\right),\\ F_{i,j-1}^{I}\cdot t\left(I_{i},I_{i}\right) \end{array}\right.$$

$$F_{i,j}^{D}=sum\left\{\begin{array}{c} F_{i-1,j}^{M}\cdot t\left(M_{i-1},D_{i}\right),\\ F_{i-1,j}^{D}\cdot t\left(D_{i-1},D_{i}\right) \end{array}\right.$$

Notes:

- The result of the Forward algorithm is a ratio of the sum, over all possible alignments, of the probability of observing T under the assumption of relationship to Q, divided by the probability of observing T under a random model. The log of this ratio is a score, and the E-value of an alignment can be computed based on how this score relates to the distribution of scores for alignments involving random sequences (see Eddy, 2008).
- This recurrence is similar to the Viterbi recurrence (Viterbi, 1967) for finding the highest-probability alignment; it differs in that it sums the values of alternate paths, rather than selecting the maximum probability path. Viterbi is essentially equivalent to the scheme used in Smith-Waterman, BLAST, MMseqs2, DIAMOND, and others (Durbin et al., 1998; Frith, 2020).
- This description addresses only the core model and assumes global alignment; local alignment, and additional states, require straightforward modifications to the recurrence, see Eddy (2008).
- The recurrence involves calculation of the products of probabilities, and can suffer from numerical underflow. The Viterbi (max) method avoids underflow by performing all computations in log space. This is not possible for the Forward algorithm, due to the fact that it adds probabilities. This is often addressed by moving values in and out of log space (supported by fast approximation of $log\left(p_{1}+p_{2}\right)$; this is the method used in nail’s implementation. Some implementations achieve further acceleration by scaling values directly in order to avoid conversion to log space entirely (Eddy, 2011).
- Though the recurrence suggests recursive function calls, the matrix can be computed by filling a table in an ordered fashion, due to the ordered local dependencies of computations. This is usually performed in row-major order (filling from upper left to lower right, one row at a time), though dependencies allow for other orders, such as filling in sequential anti-diagonals (Ropelewski et al., 1997), as is done in nail.

The Forward algorithm computes a measure of support for the relationship between T and Q, but does not directly produce a specific alignment between the two. One important byproduct of the calculation is that each (i,j) cell in the *Forward* matrices represents the probability of all alignments *ending* in the corresponding state, having accounted for the first j letters of T and the first i positions of Q. A common followup to Forward is to perform the same sort of computation in reverse, filling in tables from lower-right to upper-left based on an inversion of the recurrence for Forward. This *Backward* algorithm computes, for each cell, the probability of all alignments *starting* at $t_{j}$ and model position i. The Forward and Backward matrices can be combined (Durbin et al., 1998) to produce a posterior probability that each cell is part of the correct alignment. This posterior probability matrix can serve as the basis of an alignment with maximum expected accuracy (Holmes and Durbin, 1998; Durbin et al., 1998). We omit details, as they are not required to understand the work here, but note that typical calculation of each of these matrices is performed across the full quadratic alignment space.

### Efficient search for high-probability cloud in Forward/Backward matrices

The Forward/Backward computation described above captures the total probability of all possible alignments, and in doing so, fills in multiple matrices with quadratic size (the product of the lengths of T and Q). nail improves computational efficiency with a heuristic that exploits the fact that this is usually overkill – most possible alignments have such low probability that excluding them from computation has no relevant impact on the overall sum of probabilities (see Figure 1). nail’s sparse matrix approach aims to identify which matrix cells contain non-negligible probability, and limit calculations to touch only those cells. Doing so minimally impacts computed scores and resulting sequence alignments, while substantially reducing the total computation. In this section, we describe a heuristic approach for achieving this goal. The method, which we call *Cloud Search*, resembles the well-known X-drop algorithm used in maximum-score alignment methods such as BLAST (Altschul et al., 1990). nail begins with a seed that provides guidance on where high-probability cells are likely to be found, then expands a search forward and backward across the matrices for a cloud of cells around this seed that appear to contain essentially all relevant probability mass. This constrained space is then used as the basis for all downstream analysis.

#### Cloud Search by pruned anti-diagonal completion

**Figure 5.**
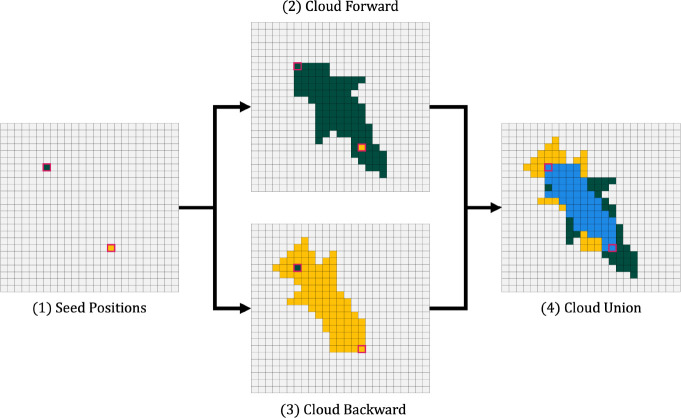
Cloud Search. In this schematic representation of Cloud Search: (1) An alignment from MMseqs2 is used as the source of begin- and end-points (green and yellow; these could come from any source). (2)Calculation is performed in the forward direction (moving down and to the right) from the begin point by filling in one anti-diagonal at a time, pruning each diagonal in from the ends based on score-dropoff conditions; this typically extends beyond the provided end point. (3) A similar flood fill pass is performed in the reverse direction starting from the provided end point, moving up and to the left. (4) The union of the two resulting spaces is identified as the sparse cloud.

The method proceeds as follows:

- Cloud Search is initiated with a pair of alignment matrix cells, *begin* and *end*. As currently implemented, this pair is taken from an MMseqs2 alignment between Q and T (Figure 5: (1)) – the first and last positions of the alignment specify the begin cell $\left(i_{b},j_{b}\right)$ and end cell $\left(i_{e},j_{e}\right)$. In principle a cell pair could be produced by some other seed finding approach, and could be initialized by more than one such pair of begin/end cells.
- Cloud Search flood-fills the matrices forward (down and right) from the *begin* cell, extending out until pruning conditions are reached – Figure 5: (2). After initializing $F_{i_{b},j_{b}}^{M}=F_{i_{b},j_{b}}^{D}=F_{i_{b},j_{b}}^{I}=0$ (green cell in upper left), neighboring cells down and right of $\left(i_{b},j_{b}\right)$ are computed in anti-diagonal fashion, first filling the two cells $\left(i_{b+1},j_{b}\right)$ and $\left(i_{b},j_{b+1}\right)$, then the three cells below these, and so on. Based on the recurrence, each cell on one anti-diagonal pushes values to recipient cells in subsequent anti-diagonals; based on this push-based transfer of information, the only cells touched on one anti-diagonal are those that are reachable from some active cell on the previous two anti-diagonals. Beginning from $\left(i_{b},j_{b}\right)$, all reachable anti-diagonal cells are computed and retained, until the anti-diagonal achieves length γ(default: 5). After this, when an anti-diagonal has been computed, two pruning conditions are applied to constrain expansion of search space.
  - Once all values in an anti-diagonal d have been computed, the maximum value for that anti-diagonal is captured as ${max}_{d}\;$. All cells with $F_{i,j}^{M}\geq {max}_{d}\;-\alpha$ are retained, and others are pruned. Scores at this point are captured in *nats* (natural logarithms), with default α=12, so that this effectively prunes cells on an anti-diagonal that have probability that is ~1 million-fold lower than the most-probable cell on that anti-diagonal.
  - As flood fill continues, the overall best-seen score across all computed anti-diagonals is captured as ${max}_{o}\;$. Any cell with score $F_{i,j}^{M}<{max}_{o}\;-\beta$ is pruned. With a default β=20, this prunes cells with ~1 billion-fold reduction from the best seen overall value (this is analogous to X in the X-drop heuristic). When all cells in an anti-diagonal are pruned, the flood fill stops.
  Pruning is performed based entirely on values stored in the Match state matrix $F^{M}$, and all scores are maintained in log space. The result of this phase is a set of cells expanding down and right from $\left(i_{b},j_{b}\right)$, schematically represented as dark green cells in Figure 5: (2). This cloud of cells typically remains in a fairly tight band around the maximum probability (Viterbi) path. Importantly, this cloud search approach typically extends well beyond the initial *end* cell $\left(i_{e},j_{e}\right)$, meaning that a conservative selection of initial points does not constrain the Forward cloud search.
- After the Forward Cloud Search phase, a similar Backward pass is performed, beginning at $\left(i_{e},j_{e}\right)$, and flood filling as in the previous stage, but up and to the left (Figure 5: (3); yellow cells).
- Cloud Search concludes by selecting the union of the Forward and Backward clouds (Figure 5: ‘Cloud Union’). This establishes a set of cells that hold a non-negligible expansion around the range bounded by the initiating cells $\left(i_{b},j_{b}\right)$ and $\left(i_{e},j_{e}\right)$.

#### Linear space requirement for computing Cloud Search

The forward and reverse passes of Cloud Search can be computed in linear space, using a 3 by m matrix, in which each row holds the dynamic programming values computed along one anti-diagonal. In general, the $n^{th}$ anti-diagonal, $d_{n}$, is assigned to row n mod 3, and each column in the cloud matrix C corresponds to a column in the implicit DP matrix. For a given matrix cell $F_{i,j}$, its anti-diagonal is given by $d_{n}=i+j$. Then, the value is stored in the cloud matrix at row (i+j) mod 3, column j. Modifications to the recurrence equations follow naturally.

**Figure 6.**
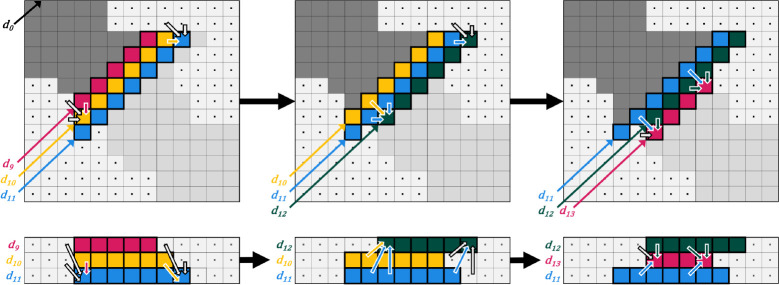
Example anti-diagonal access pattern. This example shows the implicit DP matrix (top) and the state of the cloud matrix (bottom) when anti-diagonals $d_{11}$ (left example), $d_{12}$ (middle example), and $d_{13}$ (right example) are being filled during the forward pass of Cloud Search. In both representations, the data dependency patterns are shown with arrows. Note that in the implicit DP matrix, the dependencies follow the same patterns at each step, but, in the cloud matrix, the relative positioning (in memory) of the dependencies changes.

During the forward pass of Cloud Search, the values computed along anti-diagonal $d_{n}$ depend on the values computed along the previous anti-diagonals $d_{n-1}$ and $d_{n-2}$. The cloud matrix access pattern satisfies those dependencies: the values along $d_{n}$ are stored in the row that previously contained the (now retired) values of $d_{n-3}$, while the previously computed values of $d_{n-1}$ and $d_{n-2}$ remain available. Similarly, during the reverse pass, the values along $d_{n}$ are stored in the row previously containing the values of $d_{n+3}$ with the values on $d_{n+1}$ and $d_{n+2}$ retained. Figure 6 gives an example of the cloud matrix access pattern during a forward pass of Cloud Search.

Once an anti-diagonal has been computed and pruned, the positions (in the implicit complete matrix) of its lower left and upper right cells are stored; these two cells describe an anti-diagonal cloud bound: each cell along the anti-diagonal between the two bounding cells is included in the sparse cloud. In this fashion, the cloud bounds are stored in linear space, with at most 4m values (two (i,j) pairs per anti-diagonal) describing a cloud that spans the full width and height of the complete matrix.

#### Cloud union, trimming, and reorientation

After completing the forward and reverse passes of Cloud Search, the union of the two clouds is taken, as shown in Figure 5: (4). This is done by iterating across the forward and reverse bounds from the left-most anti-diagonal present, $d_{\text{start}}$*,* to the right-most anti-diagonal present, $d_{\text{end}}$, and producing a new bound at each $d_{n}$ by combining the two bounds such that the resulting anti-diagonal covers the ranges of both the forward and reverse anti-diagonals. On anti-diagonals for which there is exclusively a forward or a reverse bound, the union step simply uses that bound.

It is possible for the clouds identified by the forward and reverse pass to not intersect; this is typically caused by a region of very low homology that Cloud Search does not pursue. In such cases, nail discards the search clouds and defaults to filling in a rectangular DP matrix bounded by the start and end seed positions. In our experience, these situations are exceedingly rare, occurring in less than 0.0001% of alignments of true positive sequences in our benchmarks.

##### Cloud trimming:

**Figure 7.**
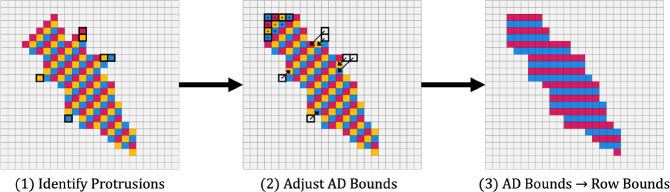
Cloud Search.

The union of the forward and reverse clouds typically results in a cloud shape with protrusions along the edges of the cloud, as shown in Figure 7: (1). The protrusions contain cells that either (a) can’t include probability from paths originating in the first anti-diagonal in the cloud, or (b) can’t propagate probability along a path toward the last anti-diagonal in the cloud. In other words, in sparse FB, the cells in such protrusions either contain a likely negligible amount of probability, or do not contribute to the total probability captured at the end of FB. Additionally, certain protrusions cause the cloud to have gaps between contiguous runs of included cells in one row of the matrix. Both classes of protrusions slightly complicate the process of reorienting the anti-diagonal-based cloud bounds into row-based cloud bounds (discussed next in this section), and row-gap-inducing protrusions dramatically complicate the bookkeeping involved with a sparse matrix data structure (discussed in the section ‘Sparse matrix organization’). Because they can not be involved in both Forward and Backward paths without passing through pruned cells, they can be safely removed from the cloud.

To remove these protrusions, we run a simple linear time algorithm that makes both a forward and reverse pass iterating across each bound in the cloud union. Pseudocode for the algorithm is given in Algorithm 1, and a visual representation can be found in Figure 7: (2).

##### Cloud reorientation:

Although the Cloud Search computations may be performed anti-diagonal by anti-diagonal, we reorient the anti-diagonal-based cloud bounds into row-based cloud bounds (primarily in preparation for a future nail implementation that will implement the J state used in HMMER3 to support multi-domain matches (Eddy, 2008). Reorientation is performed using a simple linear time algorithm that iterates across the trimmed cloud union bounds. Pseudocode for the algorithm is given in Algorithm 2, and a visual representation can be found in Figure 7: (3).


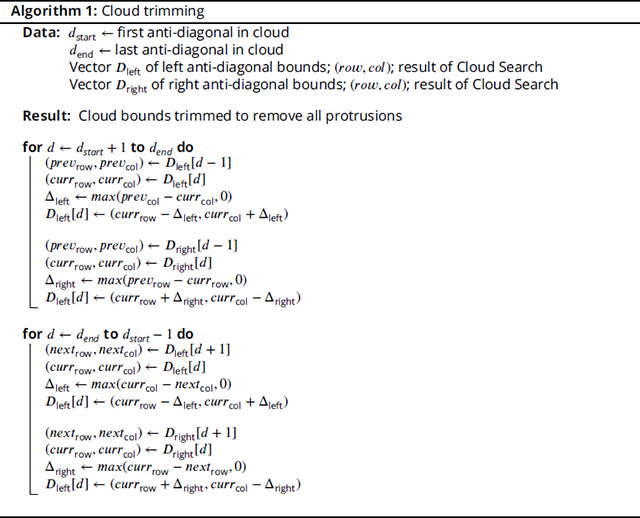



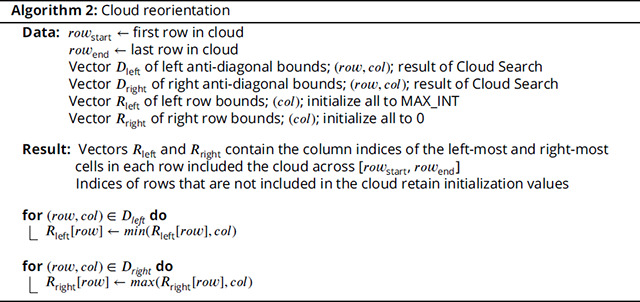


### Sparse Forward/Backward to recover score and alignment

With the cloud of non-negligible alignment matrix cells in hand, it is possible to compute an approximation of the full Forward/Backward alignment algorithm by filling in only cells in the cloud. This implicitly treats all other cells as if they carry a probability of zero.

#### Sparse matrix organization

**Figure 8.**
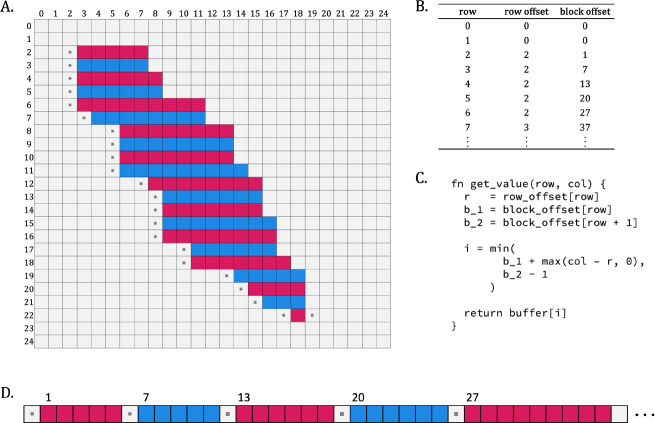
Sparse Matrix. ) Example organization of a sparse cloud into a flat array with supporting offset data, and demonstration of its use. (A) sparse cloud cells in pink/blue are supplemented with the set of padding cells (white with ▪) that ensure that any Forward/Backward calculation dependencies will refer to either a cloud or padding cell (to avoid conditionals in the DP inner loop). (B) Table of values required to compute offsets into flat array during DP recurrence computation: the row offset is the column index of the first cell in the row; the block offset is the index in the flat array of the first cell in the row. (C) Pseudocode for retrieving a value from the flat array given logical (i.e. implicit full matrix) row and column indices. The retrieval function is fast in practice, and circumvents the use of conditional logic. Note: This is a slight simplification of the actual implementation, which must support access to each of the M,I, and D values that correspond to the same logical row and column. (D) Representation of the flat array in memory. Note: the visualization has been simplified for clarity; in practice, each element in a block shown actually corresponds to a tuple of three values, one for each of the M,I, and D matrices. Similarly, each padding cell shown in the flat representation corresponds to a group of three identical padding values.

To compute a Forward/Backward approximation, the ranges defined in the row-based cloud bounds are used as the basis for creating a sparse version of each of the matrices M,I, and D. Since the row bounds describe exclusive contiguous runs of columns present in a row, we can store the M,I,D values of the entire cloud in a single flat array, with padding cells between each run of contiguous values to accommodate the data dependencies described in the FB recursion. This flat array layout is supported by a table of complementary offsets that enable rapid identification of locations in the flat array corresponding to positions in the implicit matrix (Figure 8B), with two offsets for each block of active cells. In practice, the space required to hold active and padding cells is generally only slightly larger than the number of active cells. This layout is used to allocate a sparse M,I, and D matrix in the form of an array for computing sparse Forward, another three arrays for computing Backward, and a single array for computing per-cell posterior probabilities in support of optimal accuracy alignment.

#### Sparse Forward-Backward

Computing the sparse approximation of Forward/Backward is a matter of traversing the compressed arrays in increasing order for Forward, and decreasing order for Backward, in runs defined by blocks of active cells. When filling in the sparse matrices, pad cell values are set to zero, and other cells are computed based on the standard recurrence equations, with retrieval of data via logical row and column indices supported by the function given in Figure 8C. To compute cell-wise posterior probabilities, the product of the Forward and Backward matrices are computed in the usual fashion. A Maximum Expected Accuracy alignment is identified based on these posterior probabilities (Durbin et al., 1998).

### Cloud filter, and Forward filter:

Though reduced space Forward/Backward is fast, many of the input alignment candidates will produce such a low-quality alignment that they will not end up being reported. To avoid time spent analyzing such candidates, nail performs two consecutive filters. The more robust of these is a filter applied after computing the sparse Forward score within the sparse cloud: using the sparse Forward score, a P-value is computed and alignments with P*>*1e-4 are removed (so that 0.01% of unrelated sequences are expected to pass the filter; this is similar in function to the Forward filter used in HMMER3).

Prior to computing the Forward score on the sparse cloud, nail is able to *approximate* that score using a method that we call ‘cloud filter’, which adds the sparse Forward score (starting at the begin cell) and sparse Backward score (starting at the end cell) computed during Cloud Search, approximately adjusting for score accumulated in cells shared by the two waves. This adjustment is achieved by estimating how much of the forward pass score must have been missed in the reverse pass, and vice versa. To do this, nail keeps track of the best score observed during forward Cloud Search expansion (best_fwd), and the best score observed before extending past the anti-diagonal containing end cell (best_infwd). The difference (Z = best_fwd - best_infwd) is an estimate of the part of the Forward pass’s score that is not shared by the two passes of Cloud Search. A similar value is captured during the backward pass of Cloud Search (A = best_bkwd - best_inbkwd). The total Forward score is then estimated as A + max(best_infwd,best_inbkwd) + Z; a P-value is computed for this, and only candidates with corresponding P≤1e-3 are passed on to the Forward stage.

### Bias correction, alignment boundaries, alignment:

For all downstream analyses, nail follows the methods of HMMER3, but with a sparse matrix implementation. This includes (i) estimation of the effect of composition bias on the alignment score, and corresponding score adjustment, (ii) identification of the start and end of an aligned region based on posterior probabilities captured in states that precede and follow the core HMMER3 model (HMMER’s ‘domain definition’ step), and (iii) maximum expected accuracy alignment. Resulting (bias-corrected) scores are converted to E-values as in HMMER (see Eddy, 2008). Note that bias correction depends on posterior probabilities, so bias based on sparse computation may be higher or lower than in HMMER3 – this may cause the overall (bias-adjusted) score in nail to exceed that of HMMER3.

### Test Environment

All tests were performed using 8 threads on a Linux workstation with an Intel i9–14900KF (6.0GHz boost) 24 core processor and 128GB RAM. Standard wall clock times were captured.

## Discussion

As implemented, nail demonstrates that it is possible to employ powerful Forward/Backward inference with significantly reduced time and memory requirements. Here, we highlight ways in which we expect future advances may lead to superior annotation performance.

### Better candidate seeds

nail’s dependency on MMseqs2 creates two common ways that a good alignment can be missed. In the most straightforward one, the MMseqs2 portion of the pipeline fails to find a good alignment candidate, so nail’s sparse Forward/Backward stage is never given a chance to identify the match. The fast k-mer match stage of MMseqs2 is the common cause of such misses, and is responsible for most of the sensitivity difference between nail and HMMER3. nail’s implementation makes it possible to explore development of new candidate detection options with no exposure to other parts of the algorithm. Fast and highly sensitive candidate detection may be improved through an alternative k-mer matching scheme (perhaps leveraging fast FM-index implementation as with Anderson and Wheeler, 2021), neural networks (Schütze et al., 2022), minimizer analogs (Sahlin, 2022; Joudaki et al., 2020), hardware accelerators (Anderson and Wheeler, 2023), or other methods.

### Reporting fragments or multiple domains

**Figure 9.**
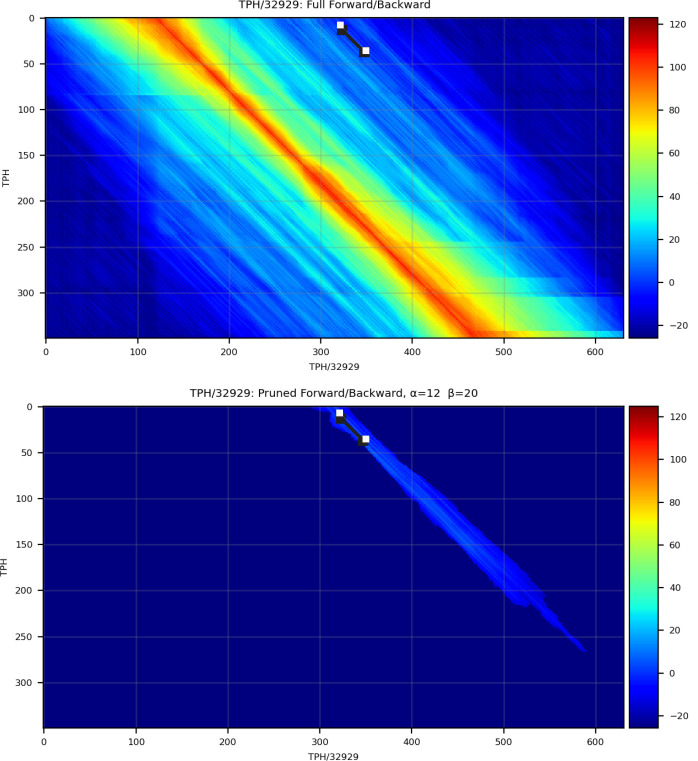
Example of MMseqs2 producing a seed outside of the dense probability cloud. Top panel shows heatmap of scores per cell in the Match State matrix for a full-DP alignment of the sequence TPHS_32929 aligned to the model for its matching family, TPH (Pfam domain PF13868). The location of the poorly placed seed produced by MMseqs2 (white line) is shown at the top center of the matrix. Bottom panel shows the sparse set of low-probability cells identified by Cloud Search based on the MMseqs-derived seed, missing the dense probability mass of the true optimal alignment. The model positions are aligned along the y-axis and the sequence positions are aligned along the x-axis.

A more subtle issue is that the current nail pipeline only analyzes the MMseqs2-sourced region with the highest score; it does not explore lower-scoring MMseqs2 matches to identify a superior Forward/Backward score/alignment. The most common impact of this will be that only a single match will be reported when there are in fact multiple hits to be found, as will be true when there are multiple copies of a query domain, or a highly fragmented sequence match. In some cases, an unfortunate MMseqs2 seed can mean that the best matching alignment is missed by nail (as in Figure 9). Mechanisms for identifying multiple good begin/end seeds, and for efficiently managing the associated sparse cloud(s), will improve nail’s completeness and sensitivity.

### Support for more complex models

nail reduces the computation workspace while retaining the core models of pHMM search. With this architecture in place, it will be possible to expand model complexity while retaining desirable run time properties. For example, it will be possible to directly incorporate models of sequence repetition (Frith, 2011; Olson and Wheeler, 2018) and sequencing error (Krause et al., 2024) for improved annotation. Furthermore, nail will also be extended to support nucleotide annotation as well as annotation of protein-coding DNA (Krause et al., 2024).

### Faster computations

The Forward/Backward recurrence calculations are modeled after the generic implementation in HMMER, with significant overhead required to support movement back and forth to log-scaled representations of odds ratios. Dynamic scaling in probability space is faster (Eddy, 2011) and should be feasible in the sparse representation described here.

## Figures and Tables

**Table 1.** Long sequence pairs

Query		Target	

Name	Length	Name	Length

TITIN_HUMAN	34,350	TITIN_MOUSE	35,213
EBH_STAAC	10,498	EBH_STAEQ	9,439
VLMS_LECSP	8,903	W4932_FUSPC	8,892
R1AB_CVH22	6,758	R1AB_BC512	6,793
HMCN1_HUMAN	5,635	HMCN1_MOUSE	5,634
RYR1_HUMAN	5,038	RYR1_PIG	5,035

**Table 2.** MMseqs2 parameters that can be altered through nail’s command line interface, along with brief descriptions of their effects (copied from mmseqs prefilter -h command). Standalone MMseqs2 internally determines a value for --k-score based on a combination of sensitivity settings and system information; this table presents the value selected by MMseqs2 for sensitive search on our benchmark tests, with kmer size of 6. nail overrides this setting with a more permissive default. Note: further reduction to --k-score will increase nail sensitivity and runtime.

Parameter	MMseqs2 sensitive	nail default	Description

--k-score	auto (88)	80	k-mer threshold for generating similar k-mer lists
--min-ungapped-score	15	15	Accept only matches with ungapped alignment score above threshold
--max-seqs	300	1000	Maximum results per query sequence allowed to pass the prefilter
